# Using virtual reality to develop emotional intelligence

**DOI:** 10.1192/j.eurpsy.2022.631

**Published:** 2022-09-01

**Authors:** K. Maslova, A. Gasimov, A. Konovalova

**Affiliations:** 1 Lomonosov Moscow State University, Faculty Of Psychology, Moscow, Russian Federation; 2 I.M. Sechenov First Moscow State Medical University, Department Of Pedagogy And Medical Psychology, Moscow, Russian Federation

**Keywords:** development of emotional intelligence, virtual reality, emotional expression

## Abstract

**Introduction:**

The development of emotional intelligence is an urgent issue of teaching people in our time. The use of a virtual reality (VR) systems for the development of emotional intelligence is a problem of modern pedagogy.

**Objectives:**

The research is aimed at studying interrelations of the level of development of emotional intelligence the manifestations of the ability to perceive and identify emotional expression demonstrated by a virtual avatar in VR CAVE system. The research is aimed at finding unusual ways to develop emotional intelligence.

**Methods:**

The study involved 55 participants aged 18 to 25 years (average age-20.38 ± 0.28), 23 of whom were men and 22 were women. During the study we diagnosed the level of development of emotional intelligence (Sergienko, Vetrova, 2009) and spatial abilities (Rimfeld et al., 2017), type of attachment to the loved one (Sabelnikova, Kashirsky, 2015), and the negotiating style of personality (Soldatova, Gasimov, 2019). In the VR CAVE system, a situation was simulated in which the subject had to detect the avatar and determine the emotional-facial expression displayed by it.

**Results:**

It was shown that the level of respondent’s emotional intelligence development does not determine the success of identifying the avatar’s emotion in VR. The success of identifying emotions depends on the level spatial abilities development. Therefore, it is assumed that in the simulated situation, the avatar is perceived as a special spatial image, and not as a full-fledged partner for interpersonal communication.

**Conclusions:**

Thus, the use of VR systems for training and development of emotional intelligence is not proven.

**Disclosure:**

No significant relationships.

